# Engineering triiodothyronine (T3) nanoparticle for use in ischemic brain stroke

**DOI:** 10.1007/s13346-012-0117-8

**Published:** 2012-12-08

**Authors:** Alexander Mdzinarishvili, Vijaykumar Sutariya, Phani K. Talasila, Werner J. Geldenhuys, Prabodh Sadana

**Affiliations:** 1 Department of Pharmaceutical Sciences, College of Pharmacy, Northeast Ohio Medical University, 4209 State Route 44, Rootstown, OH 44272 USA; 2 Department of Pharmaceutical Sciences, USF College of Pharmacy, University of South Florida, Tampa, FL 33612 USA

**Keywords:** Thyroid hormone, Brain stroke, Nanoparticles, Blood–brain barrier, Neuroprotection

## Abstract

A potential means of pharmacological management of ischemic stroke is rapid intervention using potent neuroprotective agents. Thyroid hormone (T3) has been shown to protect against ischemic damage in middle cerebral artery occlusion (MCAO) model of ischemic brain stroke. While thyroid hormone is permeable across the blood–brain barrier, we hypothesized that efficacy of thyroid hormone in ischemic brain stroke can be enhanced by encapsulation in nanoparticulate delivery vehicles. We tested our hypothesis by generating poly-(lactide-co-glycolide)-polyethyleneglycol (PLGA-b-PEG) nanoparticles that are either coated with glutathione or are not coated. We have previously reported that glutathione coating of PLGA-PEG nanoparticles is an efficient means of brain targeted drug delivery. Encapsulation of T3 in PLGA-PEG delivery vehicle resulted in particles that were in the nano range and exhibited a zeta potential of −6.51 mV (uncoated) or −1.70 mV (coated). We observed that both glutathione-coated and uncoated nanoparticles are taken up in cells wherein they stimulated the expression of thyroid hormone response element driven reporter robustly. In MCAO model of ischemic stroke, significant benefit of administering T3 in nanoparticulate form was observed over injection of a T3 solution. A 34 % decrease in tissue infarction and a 59 % decrease in brain edema were seen upon administration of T3 solution in MCAO stroke model. Corresponding measurements for uncoated T3 nanoparticles were 51 % and 68 %, whereas for the glutathione coated were 58 % and 75 %. Our study demonstrates that using nanoparticle formulations can significantly improve the efficacy of neuroprotective drugs in ischemic brain stroke.

## Introduction

Ischemic brain stroke is one of the leading causes of death and disability in major industrialized countries [1]. The deadliness of stroke is compounded by (a) a limited time window for therapeutic intervention due to a rapidly progressing pathology (approximately 3 h) and (b) lack of viable strategies to overcome the disease pathology. Thrombolytic agent recombinant tissue plasminogen activator (rt-PA) is currently the only approved agent for use in clinic. However, thrombolysis is frequently associated with reperfusion injury [2]. Substantial research efforts are now focused on neuroprotective strategies, and a number of neuroprotective agents have been suggested to improve stroke survival and outcome in preclinical studies [3].

A significant impediment to the clinical success of neuroprotective agents has been the efficient delivery of the neuroprotectant agent in high enough local concentrations to exert protective effects [4]. Blood–brain barrier (BBB) poses significant challenges to the permeation of neuroprotective agents that exert their activity at the site of action. Researchers recently have reported success in overcoming BBB using nanoparticle formulations of these drugs. In many cases, nanoparticle formulations reported significant improvement of neuroprotective activity of small molecules, endogenous cytokines and proteins [5–7].

The quest for relatively salubrious neuroprotective agents with predictable pharmacokinetic–pharmacodynamic profile has sparked interest in endogenous hormones for their activity in ischemic stroke. Estrogen and its derivatives have been a focus of tremendous interest in recent years as has been progesterone [8, 9]. Here, we have turned attention to thyroid hormone or L-3,5,3′-triiodothyronine (T3) as a neuroprotective agent.

L-3,5,3′,5′-tetraiodothyronine (T4) is a more abundant but less active form of thyroid hormone than T3. T4 undergoes peripheral deiodination by specific deiodinases to yield T3, and this process is the primary source of circulatory T3. Thyroid hormones (T3 and T4) play a crucial role in growth, development, metabolism, and cellular energetics [10]. In addition, thyroid hormones have profound effects on the central nervous system. T3 is especially required in the new neuron production and maturation during early brain development [11, 12]. Thyroid hormone deficiency in specific time windows of brain development can lead to irreversible brain damage [13, 14].

Genomic actions of thyroid hormone are mediated via the thyroid hormone receptor (TR). TR binds as a heterodimer with retinoid X receptor (RXR) on the thyroid hormone response element (TRE) in the promoter of its target genes and regulates their transcriptional activity [15, 16]. Frequently, TREs assume a DR4 (direct repeat 4) configuration in which two consensus half sites are separated by four spacer nucleotides. In the canonical mode of T3 action, TR is bound to the TRE in the unliganded state. In this state, TR is associated with co-repressor proteins. Upon ligand binding, conformational change in TR occurs and there is an increase in the affinity for coactivator proteins. Recruitment of coactivators is associated with an increase in the transcriptional activity of TR and modulation of target gene expression.

Neuroprotective property of T3 has been demonstrated previously. Non-genomic T3 activation of nitric oxide signaling and vasodilation has been suggested as a possible mechanism of neuroprotection [17]. However, other genomic mechanisms cannot be ruled out. T3 crosses the BBB via a specific transporter called monocarboxylate 8 (MCT8). However, a significant source of T3 levels in the brain is via deiodination of T4. We hypothesized that the neuroprotective potential of T3 can be maximized by administering the hormone in nanoparticulate formulations, which would lead to consistent delivery. Poly-(lactide-co-glycolide)-polyethyleneglycol (PLGA-b-PEG) nanoparticles (NP) is an excellent carrier because of their biodegradability, lower toxicity, and controllable drug release rate [18]. In our previous publication, we have demonstrated higher brain uptake of therapeutic load in rat model using the glutathione-coated PLGA-PEG NP [18]. Glutathione coating is the mechanism of enhanced brain uptake of NPs owing likely to the presence of large number of glutathione transporters at the BBB [19–21]. Using a mouse middle cerebral artery occlusion (MCAO) model of ischemic brain stroke, we demonstrate here that neuroprotective property of T3 is significantly higher in the PLGA-PEG formulations as compared to a solution of T3. Further, the activity of the hormone can be amplified using the glutathione-coated PLGA-PEG particles over uncoated nanoparticles.

## Materials and methods

### Materials

PLGA-PEG-COOH (RESOMER® RGP d 50105, copolymer ratio 50:50, PEG content 8 %) was obtained from Boehringer Ingelheim Chemicals, Inc. (Petersburg, VA, USA). Coumarin-6 and 2,3,5-triphenyltetrazolium chloride (TTC) stain were obtained from Sigma-Aldrich Co. (St. Louis, MO, USA). DMEM (Dulbecco’s modified eagle medium), fetal calf serum, and penicillin–streptomycin were purchased from Hyclone (Logan, UT, USA). Acetone and acetonitrile were purchased from VWR International (Batavia, IL, USA). HEPES, NaCl, and EDTA were received from VWR International (West Chester, PA, USA). Dual luciferase kit was from Promega (Madison, WI, USA).

### Preparation of glutathione-coated PLGA-PEG MP of T3

The NPs were prepared using a nanoprecipitation method as described previously [18]. Briefly, 5 mg T3 and 120 mg PLGA-PEG-COOH were dissolved in DMSO and mixed together drop wise into 10 ml PBS. The NPs were stirred for 2 h at 50 °C to give a final T3 concentration of 0.5 mg/ml. Then 20 mg glutathione was added to 1 ml NP solution (20 mg/ml) to get 2 % w/v glutathione coating per 1 ml NP solution and incubated at room temperature for 30 min before use. The 6-courmarin loaded NPs were prepared following the same protocol as that was described above. However, 6-coumarin (in ethanol) was used instead of T3 during the procedure. The particle size and zeta potential of uncoated and glutathione-coated PLGA-PEG NP were measured using Zetasizer (Malvern). The glutathione-coated and uncoated NPs were also characterized by transmission electron microscopy (TEM).

### Intracellular distribution of 6-coumarin-loaded MP in N2A cells

Neuroblastoma cell line Neuro-2a (N2A) was seeded with 500 μl of DMEM containing 10 % fetal bovine serum with penicillin (100 U/ml) and streptomycin (100 mg/ml) at 10,000 cells per well in an eight-well poly-d-lysine-coated chamber slide. Cells were then incubated in 5 % CO_2_ at 37 °C for 24 h to achieve 70–90 % confluent cells. The medium was aspirated and the cells were washed with PBS three times. Then 500 μl of DMEM media containing 50 μg of 6-coumarin-loaded NP 2 % glutathione (2 %w/v glutathione coating per 1 ml NP solution, concentration of NP 20 mg/ml) was added to separate wells. The cultures were then incubated in 5 % CO_2_ at 37 °C for 2 h. The media was aspirated after 2 h and cells were washed three times with PBS. DAPI was added to each well for nuclear staining and a cover slip was placed on the chamber slide. The slide was then examined by fluorescent microscopy with 20X magnification.

### In vivo brain uptake of nanoparticles

Coumarin-6 (fluorescent dye) loaded nanoparticles were prepared to monitor the uptake of the nanoparticles into the ischemic brain. Procedures identical to the preparation of T3 nanoparticles were followed with substitution of coumarin-6 for T3. The animals were injected with coumarin-6 NP (glutathione-coated or uncoated) via the jugular vein in a manner similar to T3 injection. The animals were sacrificed after 1 h. The brains were isolated and brain slices were observed under Olympus fluorescent microscope (AX70). Brain tissue was homogenized in PBS pH 7.4 and fluorescence intensity (excitation wavelength 387 nm, emission wavelength 470 nm) was measured using BioTek microplate reader.

### Luciferase assay

HepG2 cells were split at 100,000 cells/well in a 24-well plate. The cells were transfected with DR4 X 2 luciferase (500 ng), TRβ (200 ng), and Tk-Renilla luciferase (200 ng) approximately 4–5 h after seeding cells. Calcium phosphate was used to transfect cells. After about 16 h of transfection, cells were washed with PBS and media changed to serum-free media. Cells were treated with 100 nM T3 or 1, 10, and 100 nM of T3 nanoparticles (coated or uncoated). Cells were harvested 24 h after T3 or vehicle treatment and dual luciferase assay (Promega) was performed. Renilla luciferase readings were used to normalize the firefly luciferase measurement. The data was expressed as fold change in reporter expression by T3 or its nanoparticle formulations over vehicle.

### Ischemic brain stroke by transient middle cerebral artery occlusion (t-MCAO)

The experiments were conducted in accordance with NIH animal care guidelines and were approved by the Animal Care and Use Committee at Northeast Ohio Medical University. In vivo brain ischemia was induced as described in detail previously [22]. Briefly, 2- to 3-month-old (25–30 g) CD-1 male mice were subjected to 1 h of transient middle cerebral artery occlusion (t-MCAO). Mice were anesthetized with 1–1.5 % isofluorane in 30 % O_2_. Throughout surgery, temperature was maintained at 37 °C by a thermostatic blanket (rectal thermometer), and cerebral blood flow was monitored by Laser Doppler flowmetry (Moor Instruments). The skin was incised, and the left occipital and superior thyroid arteries, branches of the external carotid artery (ECA) as well as the pterygopalatine artery, branch of internal carotid artery (ICA), were exposed, electro-coagulated, and cut. After occlusion of the common carotid artery by microclip, the left ECA was ligated, coagulated, and cut distally to the cranial thyroid artery. A 15-mm monofilament nylon suture (6-0 Harvard Apparatus, Holliston, MA, USA; diameter of the heat-rounded tip 0.2–0.3 mm) was inserted into the ECA and gently advanced through the ICA until its tip occluded the origin of the MCA. Correct placement of the monofilament suture was indicated by a sudden drop of the local cortical blood flow in the left MCA territory to 15–20 % of basal flow as monitored by laser Doppler flowmetry. A sustained reduction of >80 % is indicative of successful MCAO. After successful occlusion, the monofilament was secured in place with ligature and the skin incision was closed by ligature. To induce transient occlusion, the monofilament was left in place for only 60 min. Afterwards, the monofilament was removed and the skin incision was closed by surgical clips. For drug treatment, T3 (25 μg/kg) was injected i.v. through the jugular vein. Equivalent doses of T3 nanoparticles in same volume were administered to respective mice groups. This dose of T3 elevated thyroid hormone levels but is not a hyperthyroid dose [23]. T3, T3 nanoparticles, or vehicle were injected 30 min before brain stroke induction. Vehicle (PBS) treated animals served as controls. Animal cages were placed under a medical infrared heating lamp for the first 2 h of post-surgical recovery to prevent hypothermia as described previously [24].

After 24 h, the animals were deeply anaesthetized with isofluorane (4 %) and euthanized by decapitation. The brains were quickly removed and sectioned coronally into slices of 1 mm thickness (McIlwain Tissue Chopper). Slices were incubated in a 1 % solution of 2,3,5-triphenyltetrazolium chloride (TTC) in phosphate buffered saline at 37 °C (water bath) for 15 min and then fixed by immersion in 4 % buffered formaldehyde solution. TTC stains viable brain tissue dark red based on intact mitochondrial function [25], whereas infarcted tissue areas remain unstained (white). Images were acquired by a digital camera (Nikon Coolpix), and areas of both hemispheres and the infarcted regions were quantified for each slice using image analysis software (ImageJ, NIH). We measured the areas of ischemic damage (volume) as well as brain edema (ratio). Brain edema was measured as a ratio of ipsilateral (ischemic) hemisphere in comparison to the contralateral (nonischemic) hemisphere, similar to the analyses described by previously by us and others Elliot and Jaspar [26], and subsequently developed by Sydserff et al. [27]. This allowed a direct comparison of areas of damage (volume) with brain swelling and allowed us to use each animal as its own control in addition to inter-experimental comparisons.

Analyses were done according to a protocol adapted from Rorden and Brett [28]. Briefly, an investigator blinded to the animal group allocations outlined the zones of infarction (as a clear demarcation between stained and unstained areas) as well as the left and right hemispheric contours seen in each digitized brain slice picture. Three animals were used for each experiment, and measurements were made on each slice to calculate the volume [24] of the lesion and to correct for overestimation [29] due to the effects of brain swelling where *a* = area of infarct (mm^2^), *b* = area of the infarcted (ipsilateral) hemisphere slice (mm^2^), *c* = area of the non-infarcted (contralateral) hemisphere slice (mm^2^), and *d* = brain swelling (mm^2^) = (*b* − *c*). The area (Al) of the lesion (mm^2^), corrected for swelling, was derived from the equation Al = *a* − *d*. The swelling area was designated Ae and quantified by determining the ratio between the areas of the infarcted and non-infarcted hemisphere slices, thus: Ae = *b* − *c*. Infarct volume and edema ratios of hemispheric areas were expressed as mean ± standard deviation (SD) and compared using Student’s *t* test. Values of *P* <0.05 were considered statistically significant.

## Results

**Fig. 1:**
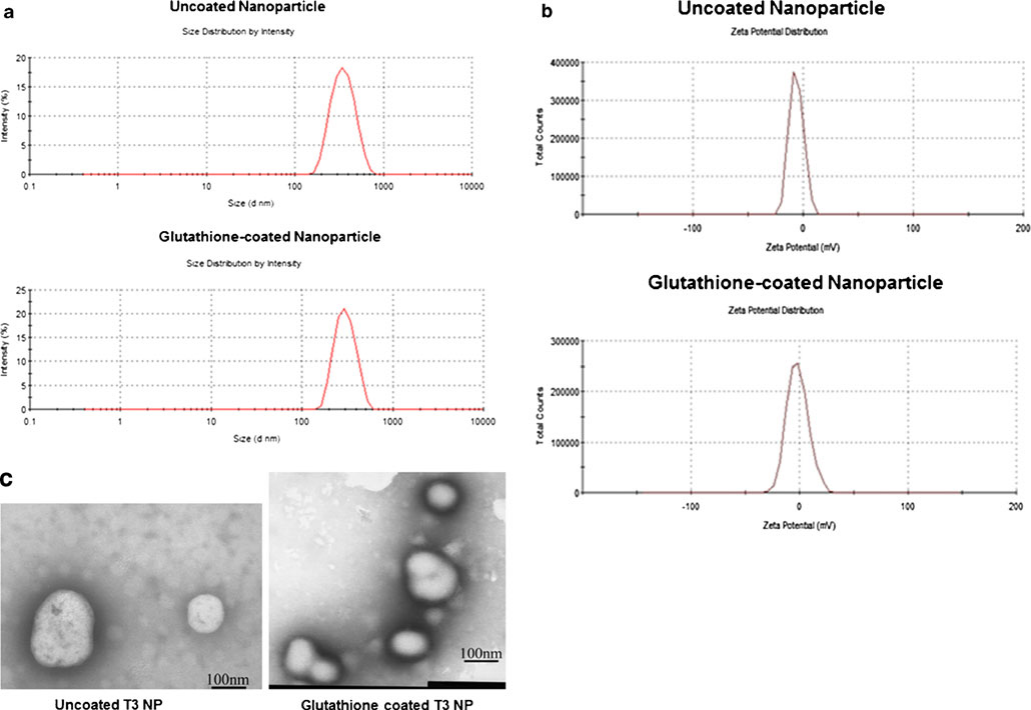
Characterization of T3 nanoparticles. **a** Determination of particle size of uncoated and glutathione-coated nanoparticles using Malvern Zetasizer. **b** Determination of zeta potential of uncoated and glutathione-coated nanoparticles using Malvern Zetasizer. **c** TEM image showing the uncoated and glutathione-coated nanoparticles

The particle size distribution and zeta potential of glutathione-coated NP containing T3 are shown in Fig. 1a (size) and 1b (zeta potential). The uncoated NP showed particle size and zeta potential of 304.6 nm and −6.51 mV, respectively while glutathione-coated NP showed particle size and zeta potential of 326.6 nm and −1.70 mV, respectively. The polydispersity indexes of both coated and uncoated NP were less than 1, which indicated the normal distribution or Gaussian distribution of nanoparticle size. In this article, the uncoated T3 nanoparticles are referred to as T3-NP and the glutathione-coated T3 nanoparticles are referred to as T3-BTNP (T3 brain targeted NP).

TEM image shows the microstructure of the nanoparticles (Fig. 1c). A size distribution in the nanometer range was observed for all the NPs in both uncoated NP and glutathione-coated NP samples. The glutathione coating is seen on the right TEM image. The TEM micrographs also reflect nearly spherical and uniform fine particles.

**Fig. 2:**
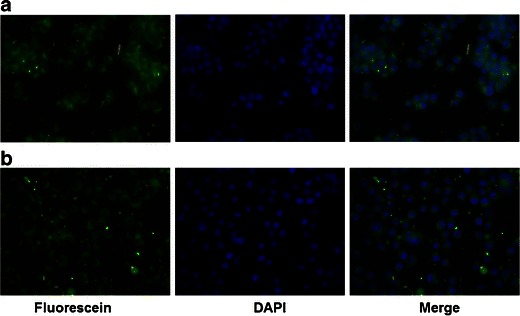
In vitro cell uptake of T3 nanoparticles. Uncoated (**a**) and glutathione-coated (**b**) coumarin-6 nanoparticles were added to Neuro2A neuroblastoma cells. Uptake of the nanoparticles was monitored using fluorescence. Uptake of coumarin-6 loaded nanoparticles was detected by fluorescein isothiocyanate filter (*green*). DAPI staining was used for nucleic acids (*blue*) and the merged overlay was generated. Magnification is 20X

To investigate the intracellular uptake of therapeutic load carried by nanoparticles, we generated courmain-6 (a fluorescent dye) loaded PLGA-PEG nanoparticles that were either glutathione-coated or uncoated. Coumarin-6 loaded NPs were added to neuroblastoma N2A cells and their uptake in the cells was monitored using fluorescence. DAPI labeling was used for nucleic acid staining. Figure 2 shows uptake of coumarin-6 in the cells as evident by the green fluorescence in the cytoplasm. Overlay of DAPI and fluorescein isothiocyanate fluorescence shows that all cells did take up the nanoparticles. Both the coated and uncoated NPs were equally well taken up by the cells and there was no evident difference in fluorescence intensity in these cells.

**Fig. 3:**
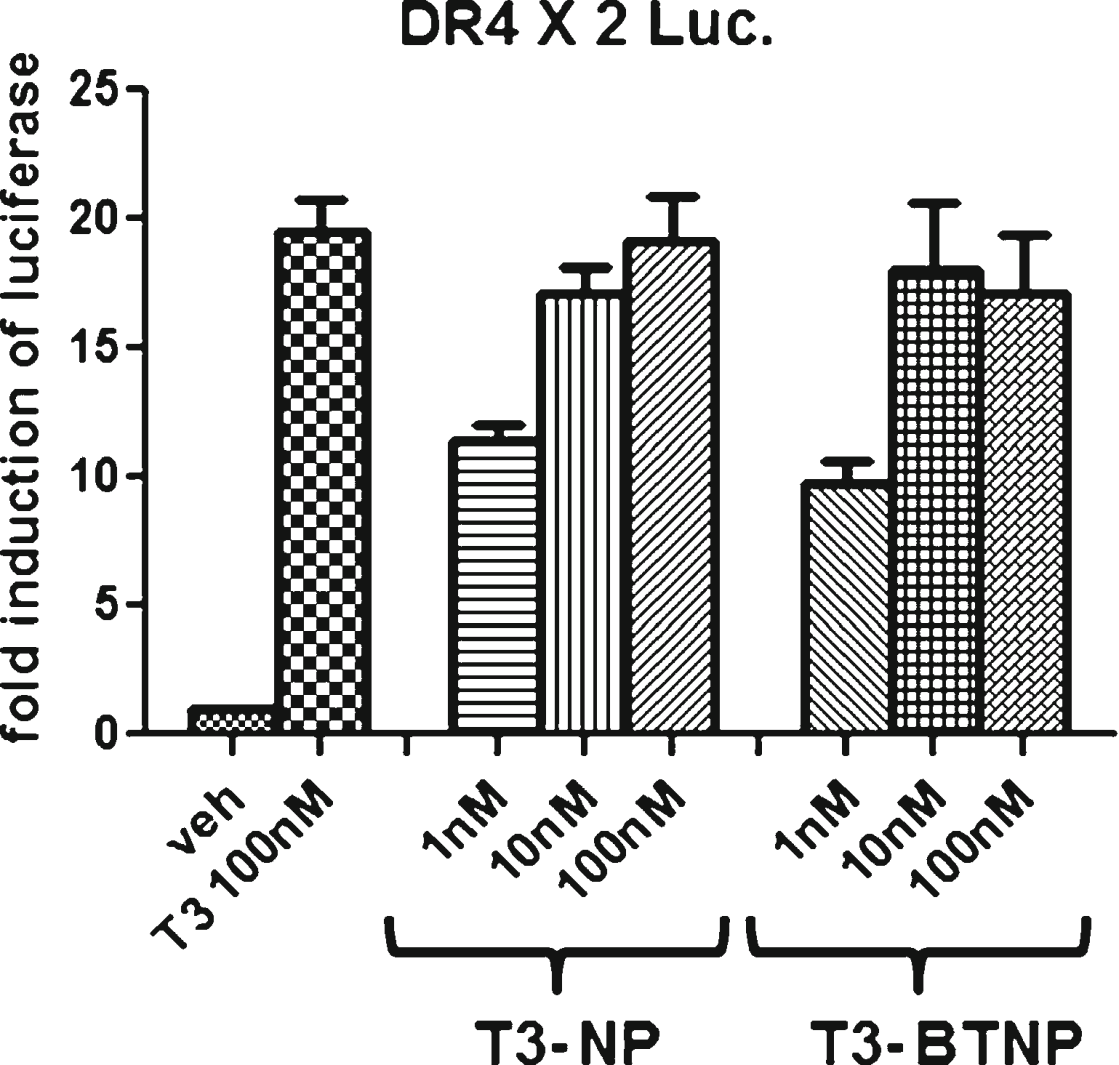
Transcriptional activity of T3 nanoparticles. HepG2 cells were transfected with DR4X2 luciferase reporter, tk-renilla luciferase, and CMV-TRβ using calcium phosphate transfection method. Cells were treated with a T3 solution or uncoated/coated T3 nanoparticles at indicated concentrations. Firefly luciferase determination was made and normalized by renilla luciferase measurements. The fold change in the luciferase induction was determined and average of three experiments performed in triplicates has been presented

Majority of physiological effects of T3 are attributed to its ability to modulate expression of target genes via TR. To evaluate whether nanoparticulate T3 retain the property to alter gene expression, we used a cell-based transcription assay. A luciferase reporter gene driven two repeats of DR4 sequence was transfected in cells followed by stimulation by either T3 solution or nanoparticulate T3. A renilla luciferase vector (tk-renilla) was also transfected as an internal control. Twenty-four hours after hormone treatment, cells were harvested and the firefly luciferase values (DR4 luciferase) were normalized to the renilla luciferase values (tk-renilla luciferase). Fold change of normalized luciferase values was determined over vehicle-treated samples. As shown in Fig. 3, the nanoparticulate T3 formulations stimulated the expression of the reporter just as robustly (10–20-fold) as a T3 solution and exhibited a dose–response relationship of this response. Thus formulation of T3 as nanoparticles does not hinder its functional activity in cells in the context of transcriptional stimulation.

**Fig. 4:**
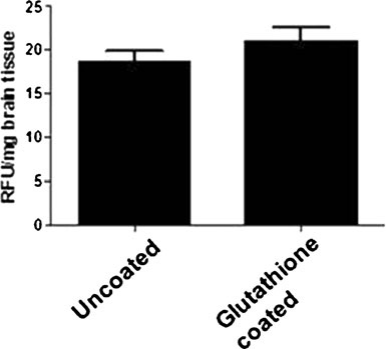
Brain tissue uptake of coumarin-6 NPs. Mice were injected with coumarin-6 NPs (uncoated or coated) i.p. for 1 h. The mice were sacrificed and brains were excised. Brain tissue was homogenized and fluorescence was determined (*n* = 3)

Next we investigated the in vivo uptake of NPs in brain. Mice were injected with coumarin-6 loaded 2 % glutathione-coated or uncoated NPs i.p. at doses similar to T3 dose used for stroke. One hour post-injection, the mice were sacrificed and the brains were excised. Both the coated and uncoated NPs showed accumulation in the brain. To quantify the levels of fluorescence, the brain tissue was homogenized and fluorescence intensity was measured. As shown in Fig. 4, both the coated and uncoated coumarin NPs were taken up in the mice brain. The coated nanoparticles showed greater uptake in the brain than the uncoated nanoparticles, though the difference was not statistically significant.

**Fig. 5:**
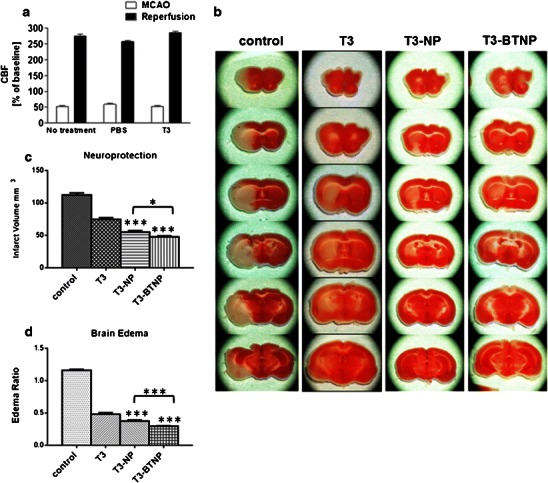
Neuroprotective activity of T3 nanoparticles in MCAO model of ischemic brain stroke (*n* = 3–6). **a** Cerebral blood flow measured by laser Doppler flowmetry. **b** TTC-stained brain slices from MCAO mouse model treated with either vehicle, T3 solution, T3 NP, or T3 BTNP. **c** Determination of tissue infarction in MCAO mouse stroke model. **d** Determination of brain edema in MCAO mouse stroke model. *p < 0.05, ***p < 0.005

Finally, we tested the nanoparticle formulations of T3 for their therapeutic benefit in ischemic stroke. Using MCAO model of brain stroke, we tested and compared the neuroprotective property of T3, T3-NP, and T3-BTNP. To ensure that brain ischemia is induced by transient MCAO, we monitored cerebral blood flow (CBF) using laser Doppler flowmetry as described previously [22]. Individual baseline signals obtained in each animal before induction of MCAO were set to 100 %. As shown in Fig. 5a, MCAO induction resulted in 80 % reduction in CBF. Animals were excluded from the experiment if the cerebral blood flow did not recover to at least 70 % of baseline by 10 min post-reperfusion.

Figure 5b shows representative brain slices of the control group as well as the T3 pre-treated group 24 h after t-MCAO. Visual inspection shows that there is a striking attenuation of the ischemic (stroke) area in those slices treated with T3 compared with controls. This attenuation was most visible in the cortical area (where the penumbra is mostly located), while the internal core (where the umbra is mostly located) was still affected by ischemia and subsequent cell death. Pre-treatment with T3 resulted in a reduction of focal ischemia by 34 % in comparison to vehicle-treated control animals (statistically significant, *P* < 0.05 versus vehicle controls) (Fig. 5c). Additionally, there was a 59 % decrease in brain swelling (edema) upon T3 pre-treatment as compared to vehicle-treated controls (Fig. 5d).

In comparison, the nanoparticulate T3 fared significantly better in our assays. T3-NP pretreatment resulted in a 51 % reduction of infarction and a 68 % reduction in brain edema as compared to vehicle-treated controls. T3-BTNP exhibited even greater efficacy in these parameters than the T3-NPs. T3-BTNP showed a 58 % reduction of tissue infarction and a 75 % reduction of brain edema than the vehicle-treated controls. The difference between T3-BTNP and T3-NP activity on these parameters was statistically significant.

Furthermore, it was seen that the increased neuroprotective activity of nanoparticulate formulations was due to greater efficacy in protecting the core area as was seen in some of the stained brain slices. While the T3 solution mainly protected the brain cortex areas from ischemic damage, the brain targeted nanoparticulate T3 is able to partially protect the core area which is most susceptible to ischemic damage.

## Discussion

In this proof-of-concept study, we have demonstrated a means to improve the efficacy of T3 as a neuroprotective agent in ischemic brain stroke. Using nanoparticles engineered to cross the BBB, we observed that the neuroprotective efficacy of thyroid hormone can be significantly enhanced. Thus far, nanoparticles have been suggested as a means to facilitate the brain delivery of BBB impermeable neuroprotectants. Our studies show that even for BBB permeable agents, the efficacy in a brain stroke model can be enhanced by formulation as fine or ultrafine particles. We show that using nanoparticles at equivalent doses, the primary pathological end-points in stroke (infarction and edema) can be improved without the need to increase the systemic exposure of T3.

We have previously demonstrated the rationale and efficacy of using glutathione-coated PLGA nanoparticles for efficient brain targeting of pharmaceuticals [18]. Findings in this study are further evidence to demonstrate the efficacy of this delivery system to increase the drug permeability to the brain. Glutathione coating on the surface allows nanoparticles to use glutathione transporters at the BBB to facilitate the entry of the therapeutic load. For BBB permeable agents like T3, we speculate that such targeted approach increases the concentration of the drug at the BBB interface and increases the probability of absorption than what would be achieved by simple diffusion or transporter mediated process. While glutathione coating can be partially responsible for greater efficacy of T3-BTNPs over T3-NPs in MCAO model, an additional mechanism can also be operative. Glutathione (GSH) serves as an important role in anti-oxidant defense and its pools are rapidly depleted upon onset of reperfusion-related brain injury in stroke [30]. Quite possibly, our delivery vehicle can help restore the normal levels of GSH in ischemic brain and offset the redox damage commonly seen in stroke. Thus, potentially there are dual mechanisms for improved efficacy of T3-BTNP over T3-NP in stroke model: increased delivery of T3 and enhancement of glutathione levels in brain.

Formulation of T3 in PLGA-PEG nanoparticles proved to be a challenge. Owing to limited solubility of T3 in acetonitrile, we had to modify our established protocol and dissolve T3 in DMSO. However, owing to lower volatility of DMSO, the particle size of the final formulation was bigger than what is usually obtained in our nano-precipitation method (50–100 nm). Conceivably, a smaller nanoparticulate T3 formulation would result in even greater enhancement of T3 efficacy in stroke by increasing its uptake and levels at the site of action. Ongoing efforts in our laboratory are currently focused on alternate means of generation of smaller nanoparticulate formulations of T3 for activity in ischemic brain stroke.

Nanoparticulate encapsulation of drug or the coating of NPs with glutathione does not impede the delivery of the therapeutic load to cells as was evident in the N2A cell study. There was no difference in coated versus uncoated MP delivery of the drug in these cells. While it is not known if the N2A cells express specific glutathione transporters, these transporters are known to exist at the interface of BBB. The fact the coated NPs demonstrated greater uptake in the in vivo uptake study while no difference was evident in the in vitro cell uptake suggests that the N2A cells lack specific glutathione transporter.

Nanoparticulate encapsulation also does not hinder the ability of the hormone to modulate gene expression. The physiological functions of T3 are predominantly reliant on its ability alter target gene expression. While non-genomic modulation of nitric oxide levels in vascular endothelial cells has been suggested as a mechanism of neuroprotective action of T3, other gene expression modulation related mechanisms are also likely [17]. Our findings show that T3 encapsulated in nanoparticles is able to induce the expression of a consensus TRE driven luciferase reporter in a dose-dependent manner and at equipotent levels as a solution of T3. Drug-release studies show that the nanoparticles release T3 at physiological pH. Our studies show that delivering T3 as nanoparticles does not alter the physiology of the hormone and functional effects of T3 are retained in the cells.

Interestingly, formulation of T3 as nanoparticles allowed the hormone to extend its neuroprotective activity to the ischemic core. While neuroprotection achieved by T3 solution was limited to the cortical areas, partial protection of the inner core of ischemic damage was observed with the T3 nanoparticles. Brain stroke is associated with debilitating neurological deficits in survivors. Hippocampus, a key region in the brain involved in cognition and behavior, was partially protected by the nanoparticles but not by T3 solution. Frequently, neuroprotective strategies cannot protect against the damage to ischemic core which is indeed the case seen with T3 solution. Our findings have highlighted the potential for enhancement of neuroprotective activity of T3 in the inner ischemic core. A likely reason for such activity could be the inherent cellular diffusibility and permeation of the nanoparticles as well as higher local concentrations of T3 achieved as a result of delivery via nanoparticles. We believe that this activity will translate into greater improvement in the functional recovery post-brain stroke when using T3 nanoparticles as opposed to T3 solution.

Several physiological parameters are changed dramatically in the infarcted area during brain stroke, e.g., pH and redox changes, all of which will likely change the physiochemical properties of a small molecule drug. Encapsulation of a drug in a nanoparticle affords an efficient means of circumventing these changes, leading to a more predictable therapeutic and patient outcome.

Of other great significance is the fact that this study is the first to report anti-edema action of thyroid hormone in ischemic brain stroke. Such activity is of immense interest clinically as rising intracranial pressure due to edema post-stroke is the prime reason for mortality in stroke. These findings suggest T3 possesses at least dual activities in alleviating stroke pathology. Our findings will further support the development of thyroid hormone pharmacotherapy in alleviating brain stroke.

Overall, this study has highlighted the feasibility and the advantage of using nanoparticle formulation of T3 over T3 solution as neuroprotective agent in ischemic brain stroke. Our findings support strong translational efforts for exploiting the neuroprotective property of T3 and suggest a means of achieving the greater efficacy of this hormone in brain stroke.
